# Resurgence of Dengue Virus Serotype 4 in Malaysia: A Comprehensive Clinicodemographic and Genomic Analysis

**DOI:** 10.3390/tropicalmed8080409

**Published:** 2023-08-11

**Authors:** Jeyanthi Suppiah, Ernie Zuraida Ali, Mohd Khairul Nizam Mohd Khalid, Sumarni Mohd Ghazali, Kok Keng Tee, Murni Maya Sari Zulkifli, Nuraisyah Ramli, Amir Hussin Adiee, Muhamad Nurrani Ramly, Fionie Robert, Sarbhan Singh Lakha Singh, Rozainanee Mohd Zain, Ravindran Thayan

**Affiliations:** 1 Virology Unit, Infectious Disease Research Centre, Institute for Medical Research, National Institutes of Health, Ministry of Health Malaysia, Setia Alam 40170, Selangor, Malaysia; murnimaya@moh.gov.my (M.M.S.Z.);; 2 Inborn Error of Metabolism and Genetic Unit, Nutrition, Metabolic & Cardiovascular Research Centre, Institute for Medical Research, National Institutes of Health, Ministry of Health Malaysia, Setia Alam 40170, Selangor, Malaysia; 3 Biomedical Epidemiology Unit, Special Resource Centre, Institute for Medical Research, National Institutes of Health, Ministry of Health Malaysia, Setia Alam 40170, Selangor, Malaysia; 4 Department of Medical Microbiology, Faculty of Medicine, Universiti Malaya, Kuala Lumpur 50603, Malaysia

**Keywords:** dengue virus serotype 4, genomics, mutation, clinical presentation

## Abstract

Dengue virus serotype 4 (DENV-4) has been the rarest circulating serotype in Malaysia, resulting in it being an understudied area. A recent observation from institutional surveillance data indicated a rapid increase in DENV-4-infected cases. The present study aimed to investigate the resurgence of DENV-4 in relation to the demographic, clinical and genomic profiles of 75 retrospective dengue samples. First, the demographic and clinical profiles obtained between 2017 and July 2022 were statistically assessed. Samples with good quality were subjected to full genome sequencing on the Illumina Next Seq 500 platform and the genome data were analysed for the presence of mutations. The effect of the mutations of interest was studied via an in silico computational approach using SWISS-MODEL and AlphaFold2 programs. The predominance of DENV-4 was discovered from 2021 to 2022, with a prevalence of 64.3% (n = 9/14) and 89.2% (n = 33/37), respectively. Two clades with a genetic divergence of 2.8% were observed within the dominant genotype IIa. The majority of DENV-4-infected patients presented with gastrointestinal symptoms, such as vomiting (46.7%), persistent diarrhoea (30.7%) and abdominal pain (13.3%). Two mutations, His50Tyr and Pro144Ser, located at the wing domain of the NS1 protein were discovered to be unique to the recently sequenced DENV-4.

## 1. Introduction

The perpetual dengue endemic in Malaysia has been the result of a constant increase in cases in a 12-decade-long (1902–2022) journey of battling dengue infections. Seemingly, an interesting pattern of dengue serotype circulation during an outbreak period has been observed, whereby major outbreaks are likely to follow switches in DENV serotypes. In other words, a predominantly circulating dengue serotype before an outbreak is replaced by another serotype that persists towards the end of the outbreak. Once the number of cases depreciates, the persistent serotype is again replaced by another serotype. The reason for the occurrence of this pattern is unclear, thus generating an epidemiological gap that needs to be addressed.

The first isolated dengue serotype in Malaysia was DENV-1, which was identified in 1954 from a minor outbreak of dengue fever in a local school in the Federal Territory of Kuala Lumpur [1]. Later, three other serotypes were discovered, indicating the circulation of a total of four serotypes in Malaysia [2]. DENV-4 marked its predominance in early 1967–1969. The beginning of the 1970s coincided with the theory of serotype shift when DENV-4 was replaced by DENV-2 as the predominant serotype. This seemed to have suppressed the existence of DENV-4 in Malaysia, as it has accounted for less than 5% of all DENV isolated in Malaysia since then [3].

As a result, DENV-4 has been an understudied serotype, characterised by the scarcity of recently published data. The available data describing the genomic evolution of DENV-4 in Malaysia is also limited. The emergence of DENV-4 genotype IIa in Malaysia was reported for the first time in 2001 [4]. This serotype was also documented in East Malaysia (Sandakan) in 2016 [5]. However, no further genomic characterization is currently available.

A recent observation from institutional surveillance data indicated a rapid upsurge in DENV-4-infected cases from 2021–2022, indicating a switch from DENV-2. Although the precise method or cause of the phenomena of serotype switching is yet unknown, there is a possibility that this was brought on by fitness selection, in which viruses continuously mutate to produce fit progeny that allow them to become more dominant than the others. This is similar to the pattern shown by the most recent epidemic of SARS-CoV-2. Using this information as a foundation, we set out to look into the clinical and demographic features of DENV-4 circulation in Malaysia from 2017 to mid-2022 and to examine the genome of the currently circulating DENV-4. We also intended to perform in silico prediction of the effect of any mutations identified in this study to elucidate the link between fitness and transmission of DENV-4.

## 2. Materials and Methods

### 2.1. Study Subjects

RNA extracted from 75 archived clinical samples that were confirmed to be DENV-4 positive by multiplex real-time RT-PCR [6] were used in this study. These samples were referred to the virology laboratory in the Institute for Medical Research, Malaysia, by sentinel hospitals for dengue molecular diagnostics from 2017 to July 2022. The sample types included serum, plasma, cerebrospinal fluid and liver biopsies.

### 2.2. Analysis of Clinical and Demographic Features

Clinical, demographic and laboratory information corresponding to the DENV-4 cases were retrieved from medical notes and our laboratory database. The data were tabulated according to categorical variables, including gender, location, antibody status and a spectrum of clinical manifestations and continuous variables, such as age and day of fever. The categorical data were described in proportions (percentage), while the continuous variables were reported as mean and standard deviation (SD).

### 2.3. Viral RNA Extraction and Full Genome Sequencing

Viral RNA extraction was performed using a QIAamp Mini Viral RNA Extraction kit (Qiagen, Germantown, MD, USA) according to the manufacturer’s instruction. The samples were subsequently subjected to DNAse treatment using a Turbo DNA-free kit (Invitrogen, Waltham, MA, USA) to eliminate any host genome interference. Full genome sequencing was performed for the samples, with a cycle threshold (Ct) of less than 30. The library was prepared using a TruSeq Stranded Total RNA kit (Illumina, San Diego, CA, USA) according to kit manual. The final library was assessed for concentration using a Qubit Fluorometer 2.0 (Invitrogen, Waltham, MA, USA). Sequencing was performed on a NextSeq 500 (Illumina, San Diego, CA, USA), with a high output run and a final loading concentration of 1.4pM.

### 2.4. Genomic Data Analysis

High-quality sequencing reads for the study subjects were obtained through BBDuk (BBTools version 38.57) trimming and filtering, after which they were subjected to assembly using MEGAHIT 1.2.8. [7]. A sequence similarity search for DENV-specific contig filtering was performed using BLASTN (version 2.9.0+) while full genome sequences were generated in FASTA format. Moreover, full genome sequences of the reference DENV-4 strains were obtained from the National Centre for Biotechnology Information (NCBI) database (https://www.ncbi.nlm.nih.gov/nuccore (accessed on 4 January 2023)). Multiple sequence alignments of the DENV-4 full genomes were executed using the MEGA-X [8] tool, and the genome sequences were studied for the presence of unique mutations. A phylogenetic tree was constructed using the Neighbor Joining algorithm. Subsequently, pairwise genetic distance analysis was performed to estimate the genetic diversity between clades and sub-clades. Additionally, the SimPlot (v3.5.1) [9] tool was utilised to check for inter- and intra-genotype recombination signals of DENV-4.

### 2.5. Modelling of DENV-4 Wild Type (WT) and the Mutant of Interest

The effects of mutations in DENV-4 NS1 protein structure identified in this study were observed through computational modelling. The X-ray crystal structure of DENV-4 NS1 is currently not available; therefore, two separate programs, SWISS-MODEL [10] and AlphaFold2 [11], were used to model the DENV-4 protein structure containing the mutation of interest in order to obtain the consensus. The X-ray crystal structure of DENV-2 NS1 was retrieved from the Protein Databank (RCSB-PDB) (PBD ID: 4O6B) and used as a template to build the wild type (WT) and mutant DENV4 NS1 model. Once the WT and mutant DENV-4 protein models were generated, energy minimisations were performed using the Swiss-PDB viewer [12] program to remove unwanted contacts. The GROMOS96 force field was used to assign atomic charges to all residues. Furthermore, for structure prediction in the AlphaFold2 program, ColabFold was used (https://colab.research.google.com/github/sokrypton/ColabFold/blob/main/AlphaFold2.ipynb#scrollTo=kOblAo-xetgx (accessed on 30 January 2023)) by applying the following setting: template_mode: pdb70, model type: AlphaFold2-multimer-v2. The WT and mutant DENV-4 protein models were then visualised using PyMol software [13]. The qualities of the predicted structure models produced by both programs were validated using ProCheck [14], ProSA-web [15] and ERRAT [16] programs to determine the stereo-chemical quality and overall quality of the protein model.

### 2.6. Structure Stability and Hydrogen Bond Analyses

The effects of the mutations were studied through structure stability and hydrogen bond analyses. Additionally, the thermodynamic stability of the WT and mutant DENV-4 protein models was analysed using FoldX [17]. The change in free energy (kcal/mol) was calculated and interpreted as follows: >3 kcal/mol was regarded as severely destabilising, 1–3 kcal/mol as destabilising and <1 kcal/mol as benign [18]. Meanwhile, hydrogen bond interactions between the native and neighbouring residues were analysed using Chimera software [19]. The relax hydrogen bond constraint was set to be within 0.4 Å, with a maximum angle of 30 degrees.

## 3. Results

### 3.1. Trend of DENV-4 Circulation in Malaysia

**Figure 1:**
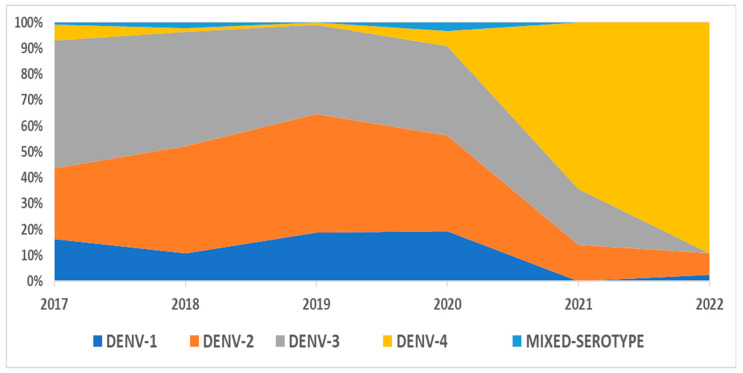
Dengue serotype distribution in Malaysia from 2017 to 2022.

**Figure 2:**
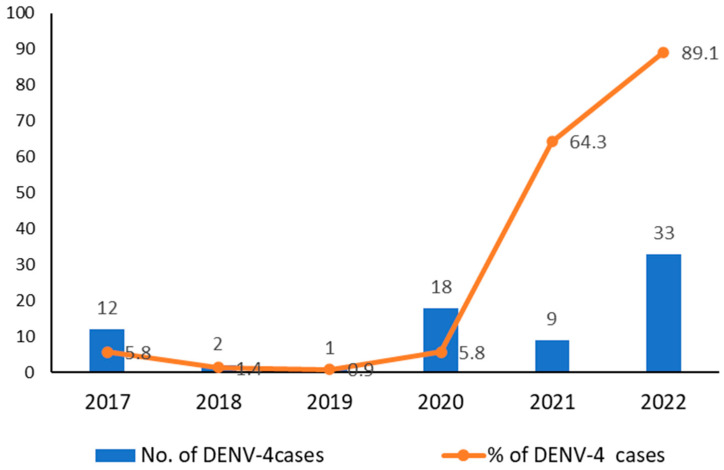
DENV-4 serotype distribution in Malaysia from 2017 to 2022.

A total of 828 dengue-positive samples, confirmed by Real-Time rt-PCR, from 2017–2020 were further classified into serotypes, as shown in Table 1. DENV-3 and DENV-2 were the predominantly circulating serotypes in 2017–2018 and 2019–2020, respectively, after which, a serotype shift was observed from 2021 onwards, with DENV-4 becoming the major circulating serotype (Figure 1). The breakdown of serotypes by states is provided in Supplement File S1. The prevalence of DENV-4 serotypes over the past 6 years, from 2017 to 2022, ranged from 0.9% in 2019 to 89.2% in 2022. Moreover, an upward trend in the DENV-4 serotype has been observed since the beginning of the COVID-19 pandemic in year 2020, as shown in Figure 2.

### 3.2. Demographic and Clinical Features of Patients Infected with DENV-4

For the overall study period (2017–2022), 75 samples positive for the DENV-4 serotype were identified, wherein the age of patients ranged from 2–80 years, with a mean of 36.1. The majority of the patients were over 18 years of age (n = 62, 82.7%), males (n = 42, 56.0%) and hailed from the central region of peninsular Malaysia (n = 37, 49.3%) (Table 2). Blood samples were collected from the patients from Day 1 to Day 12 of fever (mean = 4.3). An equal proportion of the cases (32.0%) presented with primary and secondary infections, as determined by IgM and IgG detection via dengue rapid test kits. Among the DENV-4-infected cases, 32%, 40.0% and 25.3% were diagnosed as dengue without warning signs, dengue with warning signs and severe dengue, respectively, while 2.7% succumbed to the disease (Table 3).

The most frequently reported warning signs for DENV-4 infection were vomiting (46.7%), followed by persistent diarrhoea (30.7%), lethargy (21.3%) and abdominal pain (13.3%). The remaining warning signs were observed less frequently (<10%). The most frequently reported non-warning signs were myalgia (18.7%), arthralgia (16%) and cough (10.7%). Furthermore, transaminitis and hemoconcentration were the most frequently reported dengue complications, at 16.0% and 13.3%, respectively (Table 4).

### 3.3. Genomic Characteristics of DENV-4

**Figure 3:**
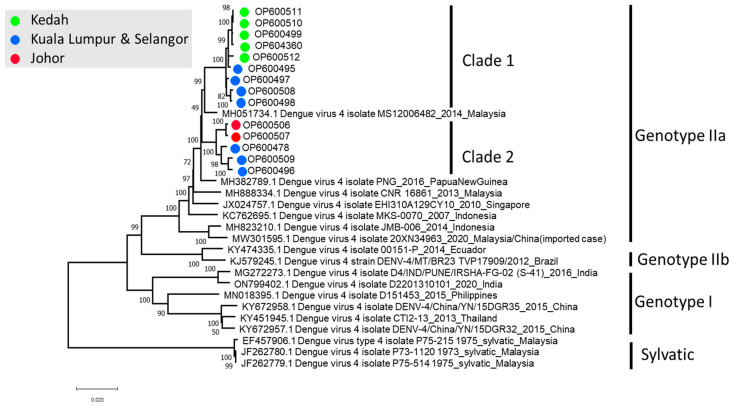
Phylogeny of the DENV-4 full genome sequences. The sequences in this study are colour-coded and can be identified as OPxxxxxx.

A total of 14 full genomes of DENV-4 were successfully sequenced in this study. This included five cases from 2020, three cases from 2021 and six cases from 2022. Phylogenetic analysis revealed that all 14 sequences belonged to DENV-4 genotype IIa. In addition, two distinct clades were observed within the genotype IIa cluster (Figure 3). Intraclade pairwise distance showed a 2.8% (2.8% ± 0.16%) genetic divergence between clades 1 and 2 of the Malaysian DENV-4 genotype IIa, although no recombination was observed. Furthermore, interclade analysis revealed minimal diversities of 0.4 ± 0.04% within clade 1 and 0.9 ± 0.07% within clade 2.

Notably, a possible geographical link was also observed between clades 1 and 2. A mixture of both clades of DENV-4 (n = 7) was found in the central region of Malaysia, consisting of states such as Selangor and the Federal Territory of Kuala Lumpur. Among samples successfully sequenced, clade 1 was largely detected in Kedah (n = 5). On the other hand, clade 2 was detected in Johor (n = 2) (Figure 3).

Two non-synonymous mutations, His50Tyr and Pro144Ser, were found in the NS1 gene, unique to the Malaysian DENV-4 genome sequences obtained in this study, while the other genes were well conserved. The mutant 50Y was detected in an overall prevalence of 57.1% of the sequenced cases in 2020 (n = 1), 2021 (n = 2) and 2022 (n = 5), while mutant 144S was detected with a prevalence of 64.3% in 2020 (n = 2), 2021 (n = 2) and 2022 (n = 5), as shown in Table 5. The impact of these mutations on the clinical presentations was not seen.

### 3.4. DENV-4 NS1 Wild Type (WT) and Mutant Models

**Figure 4:**
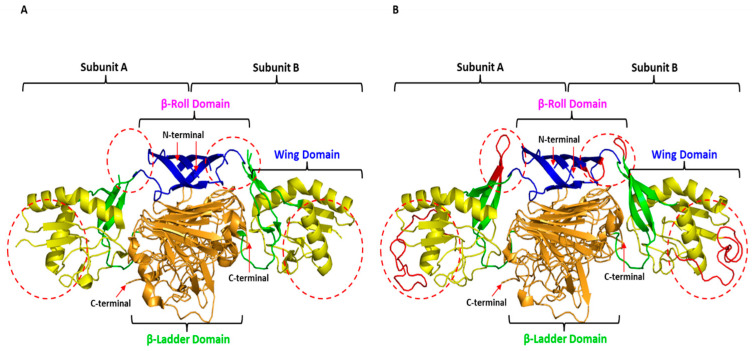
The homo-dimer X-ray crystal structure of DENV-2 NS1 (PDB ID: 4O6B) and wild type of the DENV-4 NS1 structure model: (**A**). X-ray crystal structure of DENV-2 NS1 with missing residue (red circle); (**B**). Complete wild type DENV-4 NS1 structure model developed based on the X-ray crystal structure of DENV-2 NS1. Blue colour represents the β-roll domain, yellow indicates the wing domain, green shows the connector sub-domain and orange signifies the central β-ladder domain. The red dotted circle represents unresolved regions (amino acids 7 to 11 and amino acids 108 to 128).

The mutation of interest was identified in the NS1 gene of DENV-4. Since the X-ray crystal structure of DENV-4 NS1 is currently not available, that of DENV-2 NS1 was retrieved from the Protein Databank (RCSB-PDB) (PBD ID: 4O6B) [20] and used as a template to build the WT and mutant DENV-4 models. However, this X-ray crystal structure, with a resolution of 3.0 Å, consisted of missing residues at each of the two domains. Therefore, the missing residues were added using the SWISS-MODEL program. The unresolved regions in the two domains—β-roll (amino acids 7 to 11) and wing (amino acids 108 to 128) of the DENV-2 NS1 structure were successfully constructed in the DENV-4 NS1 structure, as depicted in Figure 4. The quality of predictions by the WT and mutant DENV-4 NS1 models revealed that both structures exhibited good quality and were within the acceptance range (Table 6).

**Figure 5:**
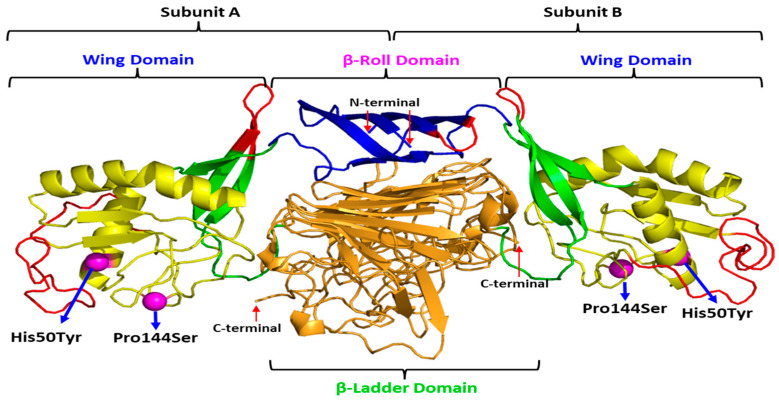
Mutations mapped onto subunits A and B of the homo-dimer wild type DENV-4 NS1.

Figure 5 depicts the position of the mutations in the DENV-4 NS1 homo-dimer structure. The mutations are mapped to subunits A and B of the homo-dimer DENV-4 NS1 structure model. Both mutations, His50Tyr and Pro144Ser, are located in the wing domain. The His50Tyr is located at α-helix 1, while the Pro144Ser is observed at the loop after β-sheet 5.

### 3.5. Mutation Effect

The effect of the mutation on the stability of NS1 is presented in Table 7. The stability of both mutant models, which were developed using Swiss Model and Alphafold, was assessed using FoldX. The prediction produced by FoldX was consistent for both mutant models. The His50Tyr mutant was predicted to be benign while the Pro144Ser mutation was predicted to be destabilizing based on the following criteria; ΔΔG value > 3 kcal/mol is severely destabilizing, 1–3 kcal/mol is destabilizing and <1 kcal/mol is neutral or benign.

**Figure 6:**
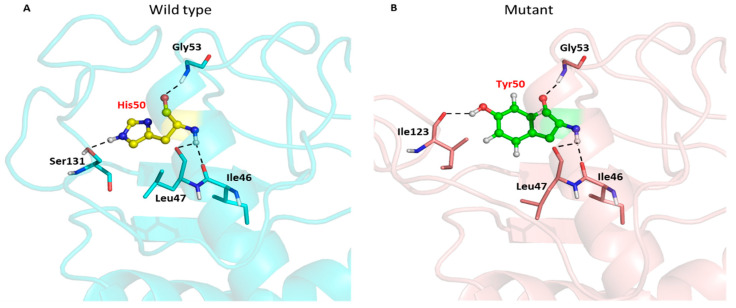
Hydrogen bond interactions between the wild type (His50) and the mutant (Tyr50) with their neighbouring residues. The wild type residue is presented as the yellow stick and sphere (**A**), while the mutant residue is presented as the green stick and sphere (**B**). Hydrogen bonds are indicated by black dotted lines. The DENV-4 NS1 structures are presented in cyan and salmon cartoons, respectively.

The substitution of histidine to tyrosine caused the loss of the hydrogen bond interaction between the backbone nitrogen atom of histidine and the oxygen atom of the serine side chain at residue 131 (Figure 6A and Table 8). However, the substituted Y50 formed a new hydrogen bond interaction with the oxygen atom of the isoleucine side chain at residue 123, as shown in Figure 6B.

**Figure 7:**
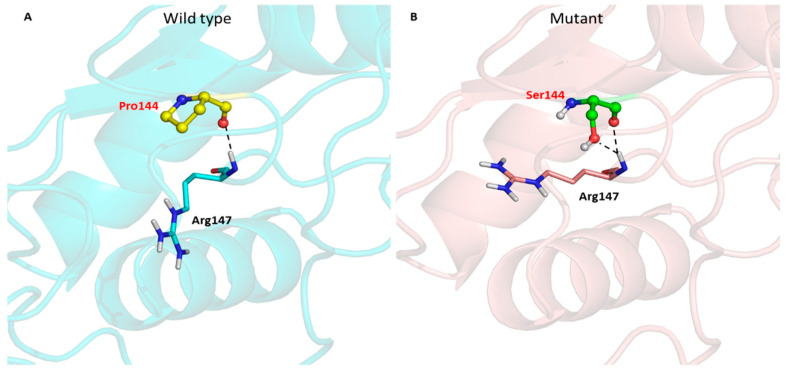
Hydrogen bond interactions between the wild type (Pro144) and the mutant (Ser144) with their neighbouring residues. The wild type residue is denoted as a yellow stick and sphere (**A**), while the mutant residue is presented as the green stick and sphere (**B**). Hydrogen bonds are indicated by black dotted lines. The DENV-4 NS1 structures are presented in cyan and salmon cartoons, respectively.

Meanwhile, the substituted Ser144 preserved the hydrogen bond interaction with arginine at residue 147 (Figure 7 and Table 8). This substituted Ser144 even introduced another hydrogen bond interaction between the oxygen atom of the serine side chain and the backbone nitrogen atom of arginine (Figure 7B).

Next, we analyzed a public dataset of genotype–fitness maps of DENV from a study reported by Dolan et al., 2021 [21] to determine the impact of the mutations observed in our study on virus evolution. The aforementioned study assessed fitness effects for all single-nucleotide variants and determined the beneficial and deleterious effect to the overall fitness of the population. The relative fitness (*w*) and its 95% confidence interval (CI) (in human cells) for His50Tyr was 0.60 (0.25–0.96) for set A and 0.45 (0.28–0.58) for set B and for Pro144Ser was 0.32 (0.13–0.53) for set A and 0.27 (0.18–0.45) for set B. Set A and B are independent passage lineage for each cell line to represent technical replicates. In mosquito cells, the *w* and CI for His50Tyr was 0.92 (0.16–1.92) for set A and 0.28 (0.19–0.44) for set B and for Pro144Ser was 0.18 (0.08–0.25) for set A and 0.15 (0.01–0.27) for set B. In set A and B, both mutations were interpreted to have deleterious effects on viral fitness in both human and mosquito cells.

## 4. Discussion

This study focuses on the circulating trend of DENV-4 in Malaysia from 2017 to 2022 to elucidate possible changes in clinicodemographic and genome profiles at points where a significant predominance was observed. National surveillance data on circulating dengue serotypes in Malaysia from 1990–2014 revealed a consistent predominance of other serotypes, such as DENV-1, 2 and 3, as compared to DENV-4 [22]. The data also showed that DENV-4 retained its existence at the background level, except for a minor increase during 2011–2012.

The findings from our study demonstrated an unprecedented predominance of DENV-4 from 2021 until 2022, while DENV-2 and DENV-3 were observed to be the major serotypes prevalent prior to these periods. A review of the most recent dengue outbreaks in neighbouring countries revealed a diverse occurrence. Singapore reported a predominance of DENV-3 from 2020–2022 [23,24], while DENV-4 had the lowest prevalence. Meanwhile, Indonesia documented a striking predominance of the DENV-4 genotype IIa during an outbreak in East Jawa from 2019–2020, with the majority of the cases presenting mild infection [25]. Additionally, the Philippines reported the emergence of the DENV-4 genotype IIa for the first time in 2015–2017 [26], whereas a national epidemic declared in 2019 was caused by DENV-3 [27]. The scenario of DENV-4 incidence in the Philippines coincided with Malaysia. In the past, DENV-4 was the least common serotype in the Philippines, where one outbreak was noted in 1964. However, a dramatic rise in DENV-4 infection cases from 2015 to 2018 was reported, which prompted a molecular epidemiological investigation. It was also determined that DENV-4 has been evolving quickly in recent years, with a significant genotype turnover to genotype IIa and the ensuing disappearance of genotype I. It was suggested that this genotype may possess higher fitness than previous strains or enhanced ability to replicate or transmit at the population level in the Philippines. This emphasises how important the genetic evolution of DENV is to establishing and maintaining transmission.

The majority of the DENV-4-infected cases during the period of our study were identified in male adults residing in the central region of peninsular Malaysia, namely the Federal Territory of Kuala Lumpur. The clinical presentations mostly indicated dengue with warning signs, such as persistent vomiting, diarrhoea, lethargy and abdominal pain. A handful of studies have shown that dengue symptoms are serotype specific [28,29]. DENV-4-infected patients commonly manifested respiratory and cutaneous symptoms [30]. Another study also reported a high likelihood of DENV-4 patients exhibiting rashes, in addition to headache and nausea [31]. In contrast, our study demonstrated a high incidence of gastrointestinal manifestations in DENV-4 infection, consistent with a study in Brazil that reported vomiting and abdominal pain as the two most frequent symptoms [32]. Gastrointestinal manifestation can be a medical concern when patients advance to bleeding and require the need for intensive care. According to one study [33], gastrointestinal bleeding complicated 4.4% of dengue infections, and according to another [34], a vast majority of patients experiencing gastrointestinal symptoms required hospitalisation. Therefore, it is critical to recognise the significance of gastrointestinal symptoms in dengue patients as soon as possible for a number of reasons, including the potential financial impact on patients and their families and the possibility that early diagnosis could prevent these patients from being admitted to the intensive care unit [35].

Similar to the findings of the present study, which showed infrequent severe cases compared to non-severe dengue, DENV-4 has rarely been related to severe clinical presentations [36]. One study, however, revealed a high prevalence of severe haemorrhagic manifestations [37]. In our study, both primary and secondary infections were identified as occurring in equal frequency during the 6-year period of analysis. However, limitations pertaining to missing antibody detection results for a number of cases were also observed. Since the data collection process was performed for surveillance purposes, it relied on the precision of the involved healthcare workers when documenting clinical information on patient forms.

In the present study, the DENV-4 full genome sequences were successfully obtained from 14 cases (average Ct 23.0 ± 5.0) between 2020 and 2022. The attempt to sequence the remaining samples was not fruitful, owing to the relatively low viral load (average Ct 31.0 ± 7.0), insufficient original samples to repeat (n = 10) and possible degradation of the RNA due to long duration of storage despite maintenance of an appropriate temperature. This resulted in small sample size and can be regarded as a limitation of this study. Nevertheless, the contribution of this study to the literature comes from its provision of more genome data, since only two Malaysian DENV-4 full genome sequences were found to be available in the NCBI database (MH888334 and MH051734) over the past years. The majority of the sequence submissions were, in fact, partial genomes, which hampered in-depth comparisons with the DENV-4 genome data prior to 2020.

The genome investigation of DENV-4 revealed the dominance of a mono-genotype and the localization of genotype IIa in Malaysia. This genotype which was first discovered in the country in 2001, was reported to confer a unique mutation at amino acid position 120 in the E gene, characterised by a substitution of serine (S) with leucine (L) [4]. The Ser120Leu mutation was found to have been well conserved in all 14 DENV-4 genomes that were sequenced in our study. A phylogenetic tree, constructed using the whole genome, revealed the existence of two clades of genotype IIa, along with a clear geographical segregation between these clades. Both clades had a distribution of cases from the central region of Malaysia, which then diverged to cases from the northern and southern regions, suggesting the possibility of the central region being the hub for the recent DENV-4 outbreak before its spread. In addition, clade 1 exhibited a stronger founder effect due to its relatively limited divergence, thus creating a high chance of widespread DENV-4 genotype IIa in the northern state of Kedah in the near future. However, this finding should be interpreted cautiously due to small sample size.

Two novel mutations in the NS1 gene from the cases in 2020–2022 were discovered to be unique to the Malaysian DENV-4 strains. Substitutions of histidine to tyrosine at amino acid position 50 of the NS1 protein and from proline to serine at position 144 were observed. As evident in this study, compared to the strains isolated in 2021–2022, the majority of DENV-4 from the year 2020 did not confer these mutations. Interestingly, this finding corresponds to the spike of DENV-4 cases from 2021 onwards. It is noteworthy that these mutations did not have a concluding pattern on the clinical outcomes of the patients. This warrants further study on the association of His50Tyr and Pro144Ser with disease severity.

The high sequence conservation of NS1 between DENV-2 and DENV-4 (>70%) (data not shown) allowed us to produce a high-quality model of mutant DENV-4. Even though protein structure analysis revealed some important characteristics of the mutations, such as its location and potential atomic interactions, the results of thermodynamic stability analysis was inconclusive. In our analysis, we found that His50Tyr mutant was predicted to be stable while the Pro144Ser mutation was predicted to be unstable. However, the positions of the mutations could influence this result because His50Tyr was located on stable alpha helices while Pro144Ser was located on a flexible loop region. FoldX is more accurate on mutations located on rigid structures and less accurate on mutations on flexible loops/disordered regions. This led us to explore the impact of these mutations on viral fitness based on a public dataset.

The NS1 protein is an enigmatic component of DENV that plays a significant role in viral replication and virion production [38]. The impact of NS1 mutations has been reported. Mutants, such as Val236Ala in DENV-2 or Trp68➔stop codon in DENV-3, were associated with decreased NS1 production and secretion, resulting in ELISA-negative diagnoses [39]. Another mutation, Thr164Ser in DENV-2 was found to decrease virus production but increased production of secreted NS1, leading to greater production of proinflammatory cytokines such as IL-1 and TNFα [40]. Pairwise growth competition assay showed that Thr164Ser has reduced fitness compared to wild-type virus [40]. The reduction in viral fitness due to mutations was a common phenomenon as it was estimated that DENV genomes have a 40–50% probability to acquire a deleterious mutation but only a 0.2–0.3% probability to acquire a beneficial mutation per replication cycle [21]. However, the consequence of having a reduced viral fitness on dengue transmission remained unclear.

The findings of this study provide valuable information on DENV-4 and its predominant genotype circulation in Malaysia. In addition, this study also provides useful information for consideration during the development of a dengue vaccine. One of the challenges to developing an effective dengue vaccine has been attributed to the characteristics of the four distinct dengue serotypes, wherein the efficacy of a vaccine depends on simultaneously neutralising high-affinity antibodies in all four serotypes. A safe and effective dengue virus vaccine needs to stimulate the neutralisation of antibodies by targeting unique sites on each of the four dengue serotypes [41]. To this end, the genome data of DENV-4 generated in our study would be a tangible reference point.

## 5. Conclusions

The present study identified DENV-4 as the predominant serotype in Malaysia from 2021 to 2022. Additionally, genotype IIa was observed to have maintained sustained circulation ever since its discovery in 2001. Although there were no significant changes in the clinical outcomes of dengue, unique mutations were discovered among the recently sequenced DENV-4. Both mutations were predicted to have deleterious effects to viral fitness, but the implications especially on dengue transmission remained elusive. Continuous surveillance of DENV is recommended to profile circulating variants in the population such as those identified in this study as well as to identify strains with epidemic potential.

## Figures and Tables

**Table 1:** Circulating dengue serotypes in Malaysia from 2017 to 2022.

Year	Number of Dengue Positive Cases Confirmed by Real-Time rt-PCR	DENV-1 n (%)	DENV-2 n (%)	DENV-3 n (%)	DENV-4 n (%)	Mixed Serotype n (%)
2017	206	34 (16.5)	56 (27.2)	102 (49.5)	12 (5.8)	2 (1.0)
2018	138	15 (10.9)	57 (41.3)	61 (44.2)	2 (1.4)	3 (2.2)
2019	116	22 (19.0)	53 (45.7)	40 (34.4)	1 (0.9)	0 (0.0)
2020	310	60 (19.4)	115 (37.1)	107 (34.5)	18 (5.8)	10 (3.2)
2021	14	0 (0.0)	2 (14.2)	3 (21.4)	9 (64.3)	0 (0.0)
2022	37	1(2.7)	3 (8.1)	0 (0.0)	33 (89.2)	0 (0.0)

**Table 2:** Demographic features of DENV-4 cases in Malaysia from 2017 to 2022.

Variable	Overall (n = 75)	
		%
Age:		
Min–Max	2–80	
Mean (SD):	36.1 (19.2)	
≤18 years	13	17.3
>18 years	62	82.7
Gender:		
Male	42	56.0
Female	33	44.0
State:		
Johor	14	18.7
Kedah	17	22.7
Kuala Lumpur	3	4.0
Melaka	2	2.7
Perak	5	6.7
Selangor	34	45.3
Region:		
South (Johor, Melaka)	16	21.3
Central (Kuala Lumpur, Selangor)	37	49.3
North (Perak, Kedah)	22	29.3

**Table 3:** Laboratory and diagnostic characteristics of DENV-4 cases in Malaysia from 2017 to 2022.

Variable	Overall	
	n	%
Day of fever:		
Min–Max	1–12	
Mean (SD)	4.3 (2.1)	
Antibody status:		
Primary infection	24	32.0
Secondary infection	24	32.0
NA	27	35.0
Diagnosis:		
Dengue without warning signs	24	32.0
Dengue with warning signs	30	40.0
Severe dengue	19	25.3
Fatal	2	2.7

NA = Data not available for either one or more components (IgM, IgG or NS1).

**Table 4:** Clinical manifestations of DENV-4 cases (n = 75) in Malaysia from 2017 to 2022.

Clinical Presentation	All (n = 75)
Warning signs:	
Persistent vomiting	35 (46.7)
Persistent diarrhoea	23 (30.7)
Lethargy	16 (21.3)
Abdominal pain	10 (13.3)
Gum bleeding	5 (6.7)
Occult bleeding	7 (9.3)
Epigastric pain	4 (5.3)
Other bleeding	2 (2.7)
Hemoconcentration	10 (13.3)
Non-warning signs:	
Myalgia	14 (18.7)
Arthralgia	12 (16.0)
Cough	8 (10.7)
Chills/Rigor	7 (9.3)
Headache	6 (8.0)
Rash	2 (2.7)
Retro-orbital pain	2 (2.7)
Severe dengue:	
Plasma leakage	5 (6.7)
Compensated shock	6 (8.0)
Decompensated shock	5 (6.7)
Complications:	
Acute kidney injury	2 (2.7)
Transaminitis	12 (16.0)
Encephalitis	3 (4.0)
Hepatitis	1 (1.3)
Multiorgan failure	1 (1.3)
Myocarditis	3 (4.0)
Respiratory distress	1 (1.3)

**Table 5:** NS1 mutation mapping for the recently sequenced DENV-4 genotype II in comparison with reference sequences.

	Sample ID	Country	Case Presentation	Collection Year	NS1 Mutations		Clade
					His50Tyr	Pro144Ser	
1	*OP600497	Malaysia	DF without WS	2020	●	●	1
2	*OP600506	Malaysia	DF with WS	2020			2
3	*OP600478	Malaysia	DF with WS	2020			2
4	*OP600495	Malaysia	DF with WS	2020		●	1
5	*OP600507	Malaysia	DF with WS	2020			2
6	*OP600496	Malaysia	Severe Dengue	2021			2
7	*OP600508	Malaysia	Severe Dengue	2021	●	●	1
8	*OP600498	Malaysia	Severe Dengue	2021	●	●	1
9	*OP600499	Malaysia	DF with WS	2022	●	●	1
10	*OP600509	Malaysia	Severe Dengue	2022	●		2
11	*OP600510	Malaysia	DF with WS	2022	●	●	1
12	*OP600511	Malaysia	DF without WS	2022	●	●	1
13	*OP600512	Malaysia	DF with WS	2022		●	1
14	*OP604360	Malaysia	DF with WS	2022	●	●	1
15	MH888334.1	Malaysia	NA	2013			
16	MH051734.1	Malaysia	NA	2014			
17	JX024757.1	Singapore	NA	2010			
18	MW301595.1	Malaysia	NA	2013			
19	KC762695.1	Indonesia	NA	2007			
20	MH823210.1	Indonesia	NA	2014			
21	MH382789.1	Papua NG	NA	2016			
22	KJ579245.1	Brazil	NA	2012			
23	KY474335.1	Ecuador	NA	2014			

DF = Dengue fever, WS = Warning sign, NA = Data not available, * = Samples sequenced in this study, • = mutation detected.

**Table 6:** Quality assessment of the predicted WT and mutant DENV-4 NS1 structures using SWISS-MODEL and AlphaFold2.

Program	WT/ Mutation	Procheck (Ramachandran Plot Statistic (%))				ProSA	Errat (%)
		Most Favoured	Additionally Allowed	Generously Allowed	Disallowed	Z-Score	Overall Quality Factor Score
SWISS-MODEL	WT	85.6	13.1	0.5	0.8	−6.67	91.2442
	p.His50Tyr	87.1	11.7	0.7	0.5	−6.7	88.2083
	p.Pro144Ser	87.1	11.7	0.7	0.5	−6.71	88.2083
AlphaFold2	WT	90.4	9.3	0	0.3	−6.95	87.1681
	p.His50Tyr	90.4	9.3	0	0.3	−7	86.9822
	p.Pro144Ser	90.4	9.2	0	0.3	−7.04	87.1681

**Table 7:** Thermodynamic stability of the predicted WT and mutant DENV-4 NS1 structures using FoldX.

Program	Mutation	FoldX (kcal/mol)
SWISS-MODEL	p.His50Tyr	−0.279206
	p.Pro144Ser	2.9665
AlphaFold2	p.His50Tyr	−0.233207
	p.Pro144Ser	3.5151

**Table 8:** Distribution of hydrogen bond interactions in the WT and mutant models.

WT/ Mutation	Donor (Amino Acid/Residue/Chain/Atom)	Acceptor (Amino Acid/Residue/Chain/Atom)	Hydrogen (Amino Acid/Residue/Chain/Atom)	Distance (Å)
WT_His50	HIS 50.A (N)	ILE 46.A (O)	HIS 50.A (H)	2.937
	GLY 53.A (N)	HIS 50.A (O)	GLY 53.A (H)	2.823
	HIS 50.A (N)	LEU 47.A (O)	HIS 50.A (H)	3.14
	HIS 50.A (NE)	SER131.A (OG)	HIS 50.A (H)	3.19
Mut_Tyr50	TYR 50.A (N)	ILE 46.A (O)	TYR 50.A (H)	2.844
	TYR 50.A (OH)	ILE 123.A (O)	TYR 50.A (HH)	2.952
	GLY 53.A (N)	TYR 50.A (O)	GLY 53.A (H)	2.823
	TYR 50.A (N)	LEU 47.A (O)	TYR 50.A (H)	3.21
WT_Pro144	ARG 147.A (N)	PRO 144.A (O)	ARG 147.A (H)	2.831
Mut_Ser144	ARG 147.A (N)	SER 144.A (O)	ARG 147.A (H)	2.88
	ARG 147.A (N)	SER 144.A (OG)	ARG 147.A (H)	3.418

## Data Availability

The full genome sequences of DENV-4 generated in this study are available in the NCBI database (accession numbers: OP600478, OP600495-OP600499, OP600506-OP600512). Other data reported in the current study are available from the corresponding author upon request.

## Supplementary Materials

The following supporting information can be downloaded at: https://www.mdpi.com/article/10.3390/tropicalmed8080409/s1, Supplement File S1: Distribution of each serotype by states from 2017-mid 2022.
