# Supplementary material for: Resurgence of Dengue Virus Serotype 4 in Malaysia: A Comprehensive Clinicodemographic and Genomic Analysis
Source: Trop Med Infect Dis. 2023 Aug 11;8(8):409. doi: 10.3390/tropicalmed8080409 (PMC10458033; doi:10.3390/tropicalmed8080409)
Supplement: Supplementary file 1 [file tropicalmed-08-00409-s001.zip › tropicalmed-2508295-supplementary.pdf]

Supplement S1

Distribution of each serotype by states from 2017-mid 2022.

States	2017						2018						2019			
	DENV 1	DENV 2	DENV 3	DENV 4	DENV 1&2	DENV 1&3	DENV 1	DENV 2	DENV 3	DENV 4	DENV 1&3	DENV 2&3	DENV 1	DENV 2	DENV 3	DENV 4
Perlis			2										0	0	0	0
Kedah		1					4	10	10		1		4	5	8	0
Penang													0	0	0	0
Perak	2	3	12	4			5	7	14	1			0	4	5	0
Selangor	16	19	34	6		1	3	28	24	1		1	13	24	13	0
Kuala Lumpur	14	26	49	2	1		2	9	7			1	0	1	0	0
Negeri Sembilan													0	0	0	0
Melaka		1							1				1	3	1	0
Johor	1	5	4					1	2				3	12	10	1
Pahang													0	3	0	0
Terengganu													0	0	0	0
Kelantan													0	0	1	0
Sarawak	1							1					0	1	1	0
Putrajaya			1				1						0	0	0	0
Sabah		1						1	3				1	0	1	0
Labuan													0	0	0	0
TotaL	34	56	102	12	1	1	15	57	61	2	1	2	22	53	40	1

States	2020								2021				2022			
	DENV 1	DENV 2	DENV 3	DENV 4	DENV 1&2	DENV 2&3	DENV 2&4	DENV 1&4	DENV 1	DENV 2	DENV 3	DENV 4	DENV 1	DENV 2	DENV 3	DENV 4
Perlis	0	0	0	0									0	0	0	0
Kedah	1	0	4	0		1						1	1	0	0	16
Penang	0	0	0	0									0	0	0	0
Perak	0	1	0	0									0	0	0	0
Selangor	21	34	33	7						1		6	0	2	0	14
Kuala Lumpur	6	3	3	0								1	0	1	0	0
Negeri Sembilan	0	0	0	0									0	0	0	0
Melaka	4	5	11	0							3	1	0	0	0	1
Johor	26	69	53	11	2	5	1	1		1			0	0	0	2
Pahang	0	1	2	0									0	0	0	0
Terengganu	0	0	0	0									0	0	0	0
Kelantan	0	0	0	0									0	0	0	0
Sarawak	0	2	0	0									0	0	0	0
Putrajaya	0	0	0	0									0	0	0	0
Sabah	2	0	1	0									0	0	0	0
Labuan	0	0	0	0									0	0	0	0
TotaL	60	115	107	18	2	6	1	1	0	2	3	9	1	3	0	33
